# Does hormonal contraception prior to in vitro fertilization (IVF) negatively affect oocyte yields? - A pilot study

**DOI:** 10.1186/1477-7827-11-28

**Published:** 2013-04-04

**Authors:** David H Barad, Ann Kim, Hala Kubba, Andrea Weghofer, Norbert Gleicher

**Affiliations:** 1 Center for Human Reproduction, New York, NY, USA; 2 Foundation for Reproductive Medicine, New York, NY, USA; 3 Department of Gynecologic Endocrinology and Reproductive Medicine, Medical University Vienna, Vienna, Austria

**Keywords:** Oral contraceptives, Hormonal contraception, In vitro fertilization (IVF), Oocyte yield, Ovarian reserve, Androgens

## Abstract

**Background:**

As oral contraceptives (OCs) suppress anti-Müllerian hormone (AMH), and hormonal contraceptives (HCs), likely, suppress functional ovarian reserve, this study was initiated to determine whether HC affect oocyte yields.

**Methods:**

We investigated in a retrospective cohort study 43 oocyte donors in 71 in vitro fertilization (IVF) cycles, evaluating anti-Müllerian hormone (AMH) and oocyte yields as reflections of functional ovarian reserve (OR). In 25 IVF cycles egg donors were on HC within one month prior to IVF, and in 46 cycles they were not. Donors, based on their HCs, were further subdivided into 12 with less, and 13 with more androgenic progestins.

**Results:**

While the three groups did not differ in age, age at menarche, BMI and AMH, oocyte yields among donors who utilized estrane- and gonane-derived (higher androgenic) HCs were lower 11.3 (95% CI 8.3 – 14.3) than either donors using no HCs 16.6 (95% CI 14.7 -18.4) (P < 0.05) or those using anti-androgenic HCs 19.0 (95% CI 12.2-25.8) (P< 0.01). Significance was maintained after adjustments for the donor age and total FSH dose used in ovulation induction.

**Conclusions:**

Even in young oocyte donors, high androgenic OC exposure appears to suppress functional ovarian reserve and oocyte yields. Since OCs are often routinely used in preparation for IVF, such practice may require reevaluation. Especially in women with diminished ovarian reserve OCs, and especially high androgenic progestin HCs, should, likely, be avoided.

## Background

Because the response to ovarian stimulation, to a large degree, depends on choice of stimulation protocols, any definition of poor response in association with in vitro fertilization (IVF) has to be considered relative. Poor response is, however, universally seen as the production of fewer than expected oocytes and embryos [1].

While by many considered a standard protocol in IVF, long agonist stimulations, because of suppressive effects on ovaries, are often considered inappropriate for poor responders [2]. Instead, hormonal contraceptives (HCs) are regularly utilized in such patients in preparation for ovulation induction [3,4]. HCs are also used to reduce ovarian cyst formation [5] and to allow convenient scheduling of cycle starts [6,7]. Results, utilizing HCs, have, however, not always been favorable [8-10]. A recent Cochrane review found that pretreatment with combined oral contraceptives (OCs) led to poorer pregnancy outcomes [11].

The primary mode of action for HCs is thought to be suppression of pituitary gonadotropin secretion, secondarily leading to suppression of ovarian function [12,13]. So-called combined HCs are combinations of estrogen (generally ethinyl estradiol, EE) and a variety of progestins. Differences among HCs, therefore, primarily reside with their progestational agents. Classical HCs have progestins, derived from estranes and gonanes, all to various degrees androgenic [14].

Synthetic progestins interact not only with progesterone receptors but also with other intranuclear steroid receptors [15]. Affinities for the various steroid receptors depend on the molecule from which a given synthetic progestin is derived [16]. In addition to progestational activity, steroid derivatives may also have androgenic, anti-androgenic, estrogenic, glucocorticoid or mineralocorticoid activities [16].

Consequently, one also has to conclude that different OCs, based on progestational content, should affect follicle maturation differently. Assuming this to be the case, different OCs could also be expected to affect functional ovarian reserve differently and, therefore, potentially oocyte yields.

Progestins may be classified into old progestins (norethisterone, levonorgestrel, gestodene) and new progestins (drospirenone, dienogest, trimegestone) [17]. Newer progestins are designed to be less androgenic. Like spironolactone, from which it derives, drospirenone functions as a competitive androgen antagonist [18,19]. Dienogest is a 19- nortestosterone derivative but it differs significantly in structure from other nortestosterone derivatives [20]. An OC containing dienogest was recently approved for use in the United States [21].

AMH is considered a good marker of ovarian aging [22]. AMH levels generally correlate well with day 2/3 FSH levels [23], and are good predictors of ovarian response to ovulation induction [24-26].

Suppressive effects of HCs on antral follicles and AMH levels have been reported. Women with polycystic ovarian syndrome (PCOS) who for 6 months took OCs, containing 35 ug of EE and 2 mg of cyproterone acetate, experienced significant decreases in antral follicles but no change in anti-Müllerian hormone (AMH) [27]. Cyproterone acetate is anti-androgenic but also suppresses gonadotropin secretion [28,29].

Whether OC utilization suppresses AMH has remained controversial, with some studies supporting such an association [30,31], and others disagreeing [32,33]. This divergence suggests that different types of OCs, containing different progestins, and maybe different utilization length, could affect results.

Oocyte donors are selected to be healthy young women, without evidence of infertility and/or abnormal functional ovarian reserve. They often use OCs or other HCs. Especially in association with utilization of the so-called “contraceptive ring” (NuvaRing™, Organon USA), which contains, and continuously releases, etonogestrel/ethinyl estradiol, we noted in some of our egg donor applicants abnormally low age-specific AMH levels. Others noted diminished numbers of small follicles amongst women using contraceptive rings in comparison to those utilizing OCs, containing EE and levonorgestrel [34].

Considering all of these observations and reports, we in here presented study decided to formally investigate the effects of progestins on follicle maturation by determining how different HCs affect oocyte yields in young oocyte donors, stimulated with standard ovarian gonadotropin protocols.

## Methods

### Population

We retrospectivley reviewed the charts of 46 oocyte donors who underwent 71 IVF cycles. Amongst those, 43 underwent at least one donation cycle, 18 two cycles, 9 three cycles, and one donor 4 cycles. At least six months elapsed between oocyte donations for donors who contributed more than one cycle to the analysis. Since our donors are all young women some of them were using HCs as their method of contraception. The choice of type of HC was made by the donor’s treating physicians outside of CHR. If used the HC used was recorded in each donor’s medical record based on the donor’s self report. Some donors changed their contraceptive status between donations and, thus, contributed in more than one category.

We routinely obtain AMH levels from donor candidates at their initial interview visit with a physician. Use of any hormonal contraceptives is allowed to continue until donor candidates are matched with a recipient, though some chose, on their own, to discontinue earlier. If more than six months elapse before a donor/recipient match, donor candidates are rescreened once matched, and before cycle start.

### Study qualifications

Donors qualified for this study if at the time of IVF cycle start less than 100 days had elapsed from their last AMH assessment.

Oocyte yields were compared between 46 cycles where oocyte donors, prior to IVF cycle, were on no HCs, and 25 cycles where donors used HCs within one month of initiation of treatment. The latter group was further subdivided into 12 anti-androgenic (11 using contraceptives with drospirenone and 1 cyproterone acetate progestin), and 13 donors with the more androgenic estrane and gonane-derived progestins. Table 1 lists the various progestins used by women in this study.

**Table 1:** Progestins in HCs of oocyte donors

**Progestin**	**Number of patients**
***Drospirenone***	11
***Cyproterone Acetate***	1
***Norethindrone***	8
***Norgestimate***	4
***Norgestrel***	1

Donors received the center’s routine IVF cycle stimulation for oocyte donors, including down-regulation with luteal phase gonadotropin releasing hormone agonist, and gonadotropin stimulation with 150–300 IU daily of human menopausal gonadotropin (hMG). Products from different manufacturers were utilized, depending on patient preference and/or insurance mandates.

Serum levels of follicle stimulating hormone (FSH) and estradiol were evaluated on cycle day 2 to 3, using the Automated Chemo Luminescence System (ACS: 180, Bayer Health Care, Tarrytown, NY). Serum AMH levels were obtained through a commercially available assay, which involves an enzymatically amplified two-site immunoassay, DSL-10-14400 active MIS/AMH ELISA (Esoterix Endocrinology, Casabasas Hills, CA).

### Clinical outcomes

Ovarian response was monitored by serial assessments of serum estradiol and transvaginal ultrasounds of ovarian follicular growth. Oocyte maturation was triggered with 10,000 IU of human chorionic gonadotrophin (hCG), when at least three dominant follicles had attained a size of 18–22 mm. Oocyte retrieval, guided by transvaginal ultrasound, was performed approximately 34 hours after hCG administration. Oocyte yields were registered in routine fashion by the embryology staff.

### Statistical analyses

Baseline characteristics of the groups were tabulated as means and 95% confidence intervals (CI) of the mean. Differences at baseline between groups were, when normally distributed, evaluated by one way analysis of variance, and by the Mann–Whitney U-test, when non-normally distributed. A generalized linear model was used to adjust for the potential confounder of age and FSH dose, used in ovulation induction, and further adjusted for repeated cycles. A P-value < 0.05 was considered significant. All analyses were carried out with SPSS software for Windows version 19.0 (SPSS Inc. Chicago, IL.)

### Ethical approval

The study underwent expedited Institutional Review Board review, since it involved only analyses of anonymous medical records. At their initial consultation, all of the center’s patients sign a consent, which allows use of their anonymous medical records for quality control and research purposes, as long as their medical records remain confidential and their identity protected. Both conditions were met. In addition, all staff with access to research data at our center, under Federal HIPAA rules, confirms their obligation to confidentiality in writing.

## Results and discussion

For the whole study group mean age was 24.2 ± 4.0 years; mean AMH 4.4 ± 2.9 ng/mL; and mean oocyte yield was 15.6 ± 7.7. Time elapsed between last AMH and IVF cycle start was 8.9 ± 3.6 weeks.

There was no difference in age, age of menarche, BMI and AMH among the three groups (Table 2). Mean AMH was non-significantly higher without HC use but did not differ significantly between the three groups. This analysis was further limited by the fact that not all study subjects had AMH values available for analysis.

**Table 2:** Donor characteristics and oocyte yields in reference to contraceptive use

	**No contraception**			**Androgenic**			**Anti-androgenic**		
	**N**	**Mean**	**95% CI**	**N**	**Mean**	**95% CI**	**N**	**Mean**	**95% CI**
***Age***	46	25.0	24.0–26.1	13	23.5	21.4–25.6	12	24.0	20.3–27.7
***Menarche***	46	13.2	12.8–13.7	13	12.8	12.1–13.5	12	13.2	12.1–14.2
***BMI***	46	19.8	19.1–20.5	13	21.1	19..5–22.7	12	19.5	18.4–20.5
***AMH***	36	4.8	3.8–5.9	12	3.6	2.4–4.9	11	4.0	2.4–5.5
***FSH Dose***	46	2276	2024–2560	13	2700	1908–3492	12	2639	2392–2885
***Oocytes***	46	16.6	14.7–18.4^*^	13	11.3	8.3–14.3	12	19.0	12.2–25.8^**^

* P < 0.05; ** P < 0.01.

**Figure 1:**
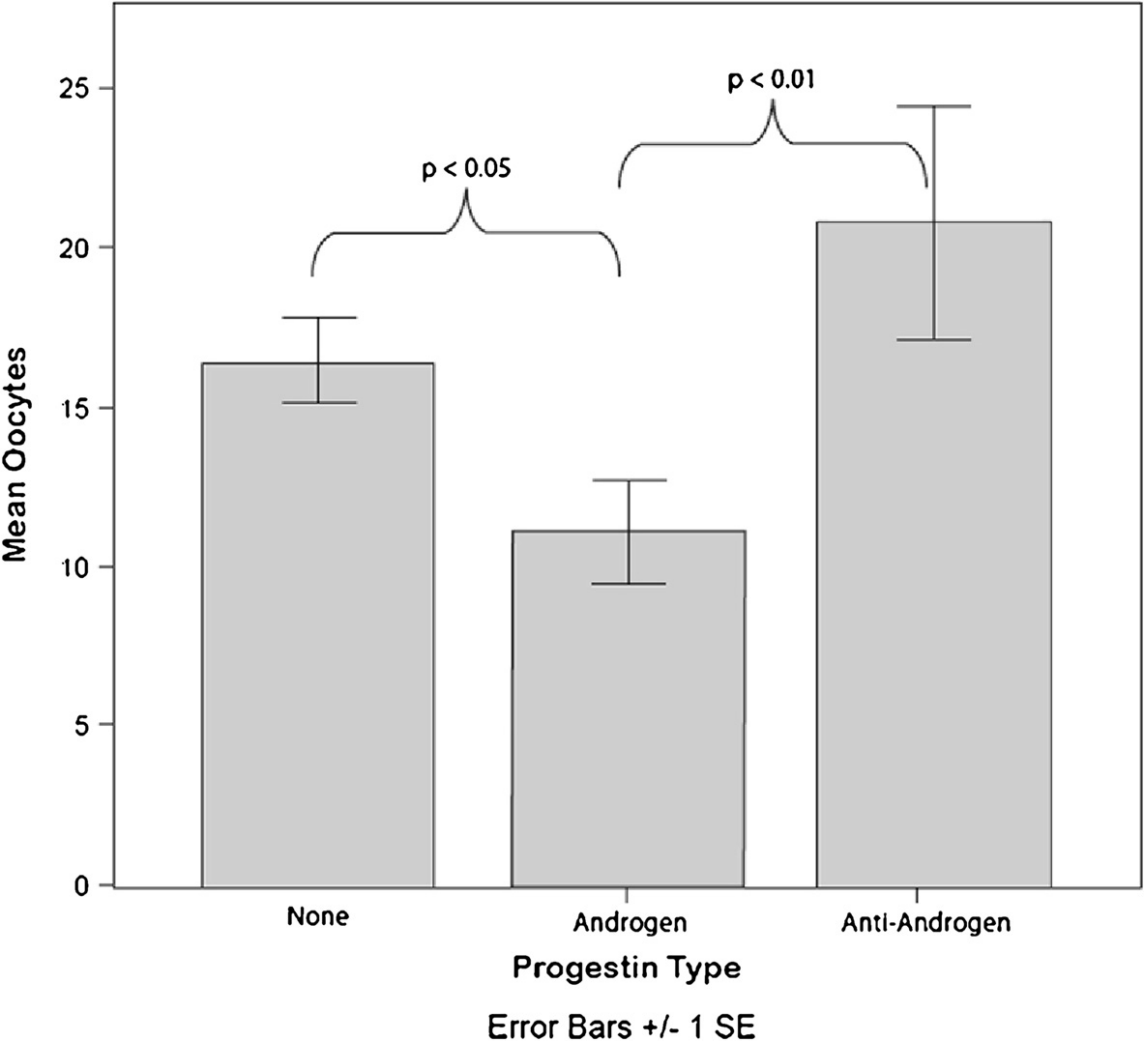
**Oocyte yields of women using androgenic hormonal contraception.** Oocyte yields of women using androgenic hormonal contraception were significantly lower than either women using no contraception or those using anti-androgenic contraceptives.

Oocyte yields among donors, who utilized more androgenic HCs, like estrane and gonane derived HCs, were lower 11.3 (95% CI 8.3 – 14.3) than those of either donors using no HCs at all 16.6 (95% CI 14.7 -18.4) (p < 0.05) or than those using anti-androgenic contraceptives 19.0 (95% CI 12.2-25.8) (p < 0.01) (Figure 1). Comparing androgenic HCs to the non-androgenic and no HC combined the omnibus test of significance was (p=0.018) after adjusting for donor age age and total gonadotropin dosage the significance was (p = 0.03).

In the introduction section of this manuscript we laid out in detail why, depending on included progestational agent, HCs should be expected to affect functional ovarian reserve in different ways. Investigating young oocyte donors, this study presents support for this concept, suggesting that recent use of HCs affects response of healthy young women to ovulation induction, resulting in significantly different oocyte yields, depending on whether HCs were utilized or not, and depending on what HCs were used. HC-use before IVF, therefore, quite apparently reduces the response to ovarian stimulation, producing lower than expected oocyte numbers. HCs, therefore, under a recently summarized consensus definition of poor ovarian response, qualify as inducers of relatively poor ovarian response to stimulation [1].

Since our center, at most, transfers two embryos in donor/recipient cycles, a diminution in oocyte yields, as here reported, likely, will not affect immediate, fresh-cycle pregnancy rates. Since total available oocytes, and especially embryos, do, however, reflect cumulative pregnancy chances, it is reasonable to conclude that any diminution of oocyte yields will also negatively affect cumulative pregnancy chances. Especially for recipients, desirous of more than one child, this may be of relevance.

The utilization of HCs in women with less favorable functional ovarian reserve than young oocyte donors, as represented by many women under standard infertility treatments with use of autologous oocytes, who may produce only relatively small oocyte yields, can, however, also be expected to affect fresh-cycle pregnancy rates since in such cycles the transfer of larger embryo numbers may be indicated.

Here presented data, while in view of a relatively small data set, still to be considered as preliminary, nevertheless, warrant a careful reevaluation of current practice patterns, involving the use of HCs in association with IVF.

### Potential mechanism for findings

As noted before, contraceptive progestins prevent ovulation through suppression of gonadotropin secretion and prevention of the LH surge [12,13]. Individual progestins, however, differ in their ability to suppress ovulation in animal models in the following declining order of potency: desogestrel > levonorgestrel > MPA > norgestimate > norethindrone [35]. Drospirenone alone, and in combination with ethinyl estradiol, suppresses ovulation but does not completely suppress follicular development [36].

We demonstrate in this study that the two anti-androgenic progestins, drospirenone- and cyproterone acetate-containing OCs, led to similar oocyte numbers as in controls, who used no HCs at all, and, therefore, to significantly larger oocyte yields than in donor who utilized more androgenic HCs.

While the estrogen component may also have a role in ovarian suppression, significant increases in AMH, antral follicle counts and ovarian volume values have been observed after discontinuation of a variety of HCs, unrelated to the estrogen dose of HCs [37].

When using OCs, significant follicular growth occurs during the seven day pill-free interval, while continuous OC use results in greater suppression of follicular activity [38,39]. This suggests that suppression of follicular growth in association with HCs may extend to antral and preantral stages of follicle maturation. Such a long-term effect may also be inferred from sometimes observed delays in resumption of normal cycles following cessation of HC. In such cases, ovarian biopsies demonstrate diffuse fibrous stroma, only primordial primary follicles and atrophic follicular cysts [40].

Considering that our group was the first to offer evidence that the weak androgen, dehydroepiandrosterone (DHEA) increases the growing follicle pool (i.e., functional ovarian reserve), resulting in higher oocyte yields and improved embryo quality [41-43], it may, on first glance, appear contradictory for this group of investigators to suggest that more androgenic progestins suppress antral follicle development. A closer look, however, reveals that such a contradiction may not really exist: Estrane and gonane progestin/ethinyl estradiol combination OCs suppress gonadotropins and, consequently, follicular development to a greater degree than drospirenone- and cyproterone acetate-containing OCs.

In early follicular development androgens work synergistically with FSH [44]. Therefore, utilizing a HC, including an androgenic progestin, will inhibit gonadotropin support for the growing follicle but maintain androgen exposure. Such a constellation may negatively affect follicular development, leading to initial androgen-driven follicle growth but, in the end, to atresia of growing follicles because of lack of FSH support.

Assuming now a normal oocyte donor without ovarian PCO phenotype, supplemented with anti-androgenic HCs, she lacks both, the androgen driven growth of small growing follicles and the growth support from FSH. Very small follicles, therefore, will fail to grow, as in above described constellation, but will, therefore, also not reach stages of atresia. Assuming discontinuation of HCs, and reconstitution of FSH support, these small follicles will, therefore, still have the ability to resume growth and development, leading to ultimately larger oocyte yields than with androgenic HCs.

Such a model of required synergism between androgens and FSH at small growing follicle stages to achieve normal follicle growth and maturation is well described in animal models [44]. It would suggest that FSH/androgen ratios at these early follicle growth stages may be predictive of later IVF cycle outcomes in humans.

### Limitations

This study’s principal limitation is the relative small number of study subjects, resulting in the description of this study as a pilot study in need of further validation. The potential importance of here first reported findings, however, warrant publication of these preliminary data to call attention to the likely suppressive effect of at least some HCs on ovarian reserve and encourage further investigations.

## Conclusions

This study, therefore, in summary, suggests that HCs, containing progestins derived from androgenic estranes and gonanes, suppress functional ovarian reserve, most likely via gonadotropin suppression, which interrupts the normal synergism between androgens and FSH at small follicle growth stages, in turn impacting oocyte yields. Since many fertility centers routinely use OCs in preparation for IVF cycles, such a practice, even in young women with normal functional reserve, appears to have negative consequences on oocyte numbers, as here demonstrated in oocyte donors.

An even more profound negative impact from androgenic HCs can, however, be expected in women with diminished functional ovarian reserve. Further investigations are needed to determine whether they should be utilized at all. At minimum, the conclusion from this preliminary study should, however, be that androgenic HCs should, likely, be avoided in women with evidence of low functional ovarian reserve.

## Abbreviations

AMH: Anti-Müllerian hormone; BMI: Body mass index; DHEA: Dehydroepiandrosterone; EE: Ethinyl estradiol; FSH: Follicle stimulationg hormone; HC: Hormonal contraceptives; hCG: Human chorionic gonadotropin; IU: International unit; IVF: In vitro fertilization; LH: Luteinizing hormone; OC: Oral contraceptives; OR: Ovarian reserve; PCO: Polycystic ovaries.

## Competing interests

N.G, A.W. and D.H.B. have in the past received research support, speakers’ honoraria and travel funds from various pharmaceutical and medical device companies, none, however, related to the subject of this paper. N.G. and D.H.B, are listed as co-inventors of two awarded U.S. patents, claiming therapeutic benefits for DHEA, and potentially other androgens, in women with DOR. Both authors have other pending patent applications, regarding DHEA, and other androgens, and the *FMR1* gene’s effects on ovaries. N.G. owns shares in Fertility Nutraceuticals, LLC, a company that offers a DHEA product. N.G. and D.H.B. are receiving patent royalties from this company. N.G. is also the owner of The CHR, where this research was conducted. Other authors have no conflicts to declare.

## Authors’ contributions

DHB and NG contributed equally to the manuscript, including study design, data analysis and writing of the manuscript, AK and DHB performed data analyses and statistical analyses, AW contributed to study design. All authors approved the final manuscript.
